# QTL Mapping of Growth-Related Traits in a Full-Sib Family of Rubber Tree (*Hevea brasiliensis*) Evaluated in a Sub-Tropical Climate

**DOI:** 10.1371/journal.pone.0061238

**Published:** 2013-04-19

**Authors:** Livia Moura Souza, Rodrigo Gazaffi, Camila Campos Mantello, Carla Cristina Silva, Dominique Garcia, Vincent Le Guen, Saulo Emilio Almeida Cardoso, Antonio Augusto Franco Garcia, Anete Pereira Souza

**Affiliations:** 1 Molecular Biology Center and Genetic Engineering, UNICAMP, Campinas, SP, Brazil; 2 Department of Genetics, ESALQ - University of São Paulo, Piracicaba, SP, Brazil; 3 CIRAD, UMR AGAP, Montpellier, Hérault, France; 4 Laboratório de Pesquisa & Desenvolvimento Plantações Michelin da Bahia, Plantações Michelin da Bahia LTDA, Igrapiuna, BA, Brasil; 5 Department of Plant Biology – Biology Institute and Molecular Biology Center and Genetic Engineering, UNICAMP, Campinas, SP, Brazil; Pennsylvania State University, United States of America

## Abstract

The rubber tree (*Hevea* spp.), cultivated in equatorial and tropical countries, is the primary plant used in natural rubber production. Due to genetic and physiological constraints, inbred lines of this species are not available. Therefore, alternative approaches are required for the characterization of this species, such as the genetic mapping of full-sib crosses derived from outbred parents. In the present study, an integrated genetic map was obtained for a full-sib cross family with simple sequence repeats (SSRs) and expressed sequence tag (EST-SSR) markers, which can display different segregation patterns. To study the genetic architecture of the traits related to growth in two different conditions (winter and summer), quantitative trait loci (QTL) mapping was also performed using the integrated map. Traits evaluated were height and girth growth, and the statistical model was based in an extension of composite interval mapping. The obtained molecular genetic map has 284 markers distributed among 23 linkage groups with a total length of 2688.8 cM. A total of 18 QTLs for growth traits during the summer and winter seasons were detected. A comparison between the different seasons was also conducted. For height, QTLs detected during the summer season were different from the ones detected during winter season. This type of difference was also observed for girth. Integrated maps are important for genetics studies in outbred species because they represent more accurately the polymorphisms observed in the genitors. QTL mapping revealed several interesting findings, such as a dominance effect and unique segregation patterns that each QTL could exhibit, which were independent of the flanking markers. The QTLs identified in this study, especially those related to phenotypic variation associated with winter could help studies of marker-assisted selection that are particularly important when the objective of a breeding program is to obtain phenotypes that are adapted to sub-optimal regions.

## Introduction

*Hevea brasiliensis* (Wild. Ex. Adr. de. Juss. Muell. Arg.) is a commercial rubber-producing species that belongs to the *Euphorbiaceae* family. The genus *Hevea* includes 11 species that are native to the Amazon region [1]. Although the growth and rubber yields of *H. brasiliensis* are optimal under hot and humid climate conditions, which prevail in its native region, it is often cultivated in dryer and colder areas throughout the world. In certain native regions, particularly South America, the devastating South American Leaf Blight (SALB) caused by the fungus *Microcyclus ulei* has become an important threat to rubber tree cultivation.

As a result of SALB, rubber production expanded during the late 1970s into worldwide, such as northeast India, the highlands and coastal areas of Vietnam, southern China and the southern plateau of Brazil [2]. All of these areas, however, exhibit various climate constraints. Although these areas satisfy most of the basic growth requirements for rubber production, they also present stressful conditions, such as low temperatures and dry periods [3]. In addition to the damage and the low growth rate caused by the cold temperatures, the production of latex is also halted for approximately 1–3 months of each year in these areas [4], [5].

Historically, the breeding of rubber trees has been based on techniques involving statistics and quantitative genetic approaches to determine the best genotypes to be used as new cultivars. The discovery of molecular genetic markers has provided new possibilities for characterizing genotypes for the purpose of identifying cultivars, analyzing genetic diversity, establishing relationships between agricultural traits and genetic factors (QTLs), and identifying genes of interest [6]–[16]. Although the genetic basis of disease resistance has been studied, less is known about the genetic basis of cold tolerance and the characteristics related to growth and plant development in the rubber tree.

Genetic maps are highly useful tools in breeding programs for many species. For perennial trees, such as *Hevea* spp., however, it is difficult to obtain homozygous inbred lines due to their extended juvenile period and inbreeding depression. Therefore, the traditional methods used to construct linkage maps for F_2_ and backcross populations cannot be used for these species [17].

Traditionally, the construction of genetic maps from a full-sib cross (or F_1_ populations) involved the double “pseudo-testcross” strategy, in which only markers that segregate in a 1∶1 ratio are considered. In the case of the rubber tree, the first genetic map was generated using a double “pseudo-testcross” strategy [9] and was obtained using several markers. This map was an important step toward obtaining a saturated genetic linkage for the rubber tree.

Considering modern markers technologies available for full sib populations, markers that segregate in 3∶1 (dominant), 1∶2:1 (codominant), and 1∶1:1∶1 (codominant) ratios, in addition to 1∶1, can be used to integrate individual linkage maps. Wu et al. [18] showed that dominant markers provide less information in linkage analyses than do codominant markers, and loci that segregate in a 1∶1:1∶1 or 1∶2:1 ratio are highly informative. Based on these findings, Wu et al. [18] proposed an alternative strategy that uses maximum likelihood methods that simultaneously estimate linkage and linkage phases. This approach circumvents several of the disadvantages emphasized by Maliepaard et al. [19] when estimating linkage phases in a separated step. This method was successfully used in sugarcane [20], [21] and a yellow passion fruit population [22].

OneMap software [23] was initially developed to facilitate linkage analyses in outcrossing species using the methodology proposed by Wu et al. [18]. Later, the software has also been updated to perform multipoint analyses based on the Hidden Markov Models [24]. This methodology allows for the analysis of a mixed set of different marker types that exhibit various segregation patterns, resulting in a genetic map that more accurately represents the polymorphisms of the cross.

Although many statistical methods have been specifically developed to perform QTL mapping in outcrossing species, the general double pseudo-testcross method is the most frequently used approach to studying the QTLs in rubber tree species [9], [25], [26].

Gazaffi et al. [27] proposed a method in which QTL mapping is performed in an integrated genetic map using Composite Interval Mapping (CIM). This method is based on a combination of different models and incorporate conditional QTL multipoint probabilities, which are more accurate and provide better results. Using this method, it is possible to infer the segregation of QTLs as well as the linkage phase between putative QTL and markers in the integrated genetic map.

In this study, we present an integrated rubber tree map saturated with simple sequence repeats (SSRs) and expressed sequence tags (EST-SSRs). We also conducted QTL mapping using CIM to determine the growth rate during the summer and winter seasons and evaluate growth characteristics (height and girth) of *H. brasiliensis*.

## Materials and Methods

### Mapping Population

The mapping population has 270 individuals derived from a cross between genotypes PB217 and PR255. The former has a high yield potential, which is expressed throughout the lifetime of its rubber production, and exhibits a low metabolic activity and a high level of sucrose in its latex vessels and the latter shows tolerance to injury and cold. The progeny were obtained through the controlled pollination of the two parental genotypes, and multiple replicates were generated by bud grafting onto rootstock (seedlings of GT1 genotype) for the field experiment.

The mapping population was planted in the Edouard Michelin Plantation (Mato Grosso state, Brazil, 17° 23′ 59.60″ S and 54° 44′ 53.93″ W, altitude 519 m) that is characterized by a sub-tropical climate with a cold and dry period that lasts four months from May to October (Figure S3). Planting was performed in 2006 using a randomized complete block design with four replicates. Each block consisted of 272 elementary plots: 1 plot for each of the 270 progeny individuals, 1 for each of the 2 parents. Each elementary plot consisted of four grafted plants with the same genotype. The statistical analysis considered data from the average of each elementary plot, i.e., average of four plants. Row and column number of each data point were annotated and included in the statistical model of the experiment that used 7.2 hectares, 40 rows and 28 columns. The field management procedures were the same as those procedures used for commercial purposes.

### DNA Extraction and Molecular Marker Analysis

Genomic DNA was extracted from 300 mg of lyophilized leaf tissue using the cetyl trimethyl ammonium bromide (CTAB) method, as described by Murray & Thompson [28]. The DNA concentrations were estimated using electrophoresis on ethidium bromide-stained agarose gels with appropriate molecular weight standards.

The polymorphisms from a total of 603 microsatellite markers from different sources were screened for both the PB217 and PR255 parents. From the total number of microsatellite markers, 425 SSR were from an enriched genomic library [13], [14], [16] and 178 were sequence tag-derived simple sequence repeat (EST-SSR) markers [29].

Two alternative techniques were used to visualize the polymerase chain reaction (PCR) products; both of these techniques required separation by electrophoresis on denaturing acrylamide gels. For visualization by silver staining, PCR was performed as described by Souza et al. [13]. For visualization by fluorescence using a LI-COR 4300 DNA analyzer, oligonucleotides with an M13-19 base extension were added to the forward primers. The PCR amplification was performed according to the methodology described by Le Guen et al. [14].

### Linkage Map

Segregation of markers was tested using the common procedure based on the Chi-square test, correcting for multiple tests. Linkage analysis was performed using OneMap software [23] version 2.0-1 using multipoint technology based on Hidden Markov Models [18]. Initially, cosegregation groups were established using an LOD score of 4.5 and a recombination fraction of 0.4. The order of the markers was obtained with algorithms *compare* (for groups with up to six markers) and *order* (for groups with more than six markers) [30], [31]. To determine the map, recombination fractions between markers were converted to centiMorgan units using the Kosambi map function [32].

### Field Data Analysis

Girth (circumference at 1 m) and height were measured in centimeters (cm) to evaluate the growth of individual rubber trees, and means were calculated based on four plants per plot. These growth characteristics were measured in April and October from 2007 to 2009, when the plants had 18, 24, 30, 36 and 42 months for each plant per plot and the average was calculated for each plot. The traits were defined as the difference in growth between October to April (summer) and April to October (winter) over two years. The values for the summer period were calculated by the subtraction of the measures obtained for 24 minus18 months (first year) and 36 minus 30 months (second year). The growth ratios for first and second year were added to obtain plant development during the summer season. Winter values were obtained by subtracting from 30 to 24 months and 42 to 36 months, and adding these two values was obtained plant development during the winter season. The total development was obtained from the subtraction measures evaluation for 42 and18 months. Six traits were considered: Total Height (TH), the height growth in the summer (SH) and winter (WH), Total Girth (TG), and the girth growth in the Summer (SG) and Winter (WG).

The statistical model used for the analysis was:
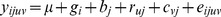
where y_ijuv_ is the growth characteristic measured for one season over two years for the i^th^ genotype in the j^th^ block, the u^th^ row and the v^th^ column; μ is the intercept; g_i_ is the random effect of the i^th^ genotype ∼N(0, V_g_); b_j_ is the fixed effect of the j^th^ block; r_uj_ is the random effect of the u^th^ row within the j^th^ block ∼N(0, V_row_); c_vj_ is the random effect of the v^th^ column within the j^th^ block ∼N(0, V_col_). Here, the usage of rows and columns (indicating the elementary plots) as random effects was an attempt to control local variation, as similarly done by Boer et al. [33] and Thumma et al. [34]; e_ij_ is the error term ∼N(0, V), assuming heteroscedasticity between blocks and no correlation among plots.

Analyses were performed using the Genstat Software [35], and the predictions of genotypic values (BLUPs) were used to perform the QTL mapping. The heritability for each trait was estimated based on the variance components from the phenotypic analysis.

The genetic correlations (r_g_) between each pair of traits were obtained using the Pearson’s correlations coefficient applied on the individual genotypic values. These correlations were tested assuming global significance level of 0.05. The analyses were done using R software (www.r-project.org).

### QTL Mapping

QTL mapping was performed using the integrated genetic map according to the approach described by Gazaffi [27]. The two traits (girth growth and height growth) were analyzed separately for each season. Briefly, the applied methodology extends composite interval mapping (CIM), as presented by Zeng [36], for an outbred scenario. The model has three genetic effects: two for additive effects (one for each parent), and one for dominance. To infer the conditional probabilities of QTL genotypes, given marker genotypes, multipoint probabilities were obtained using Hidden Markov Models.

The mapping strategy is based on two steps. First, the genome scan is performed to detect QTLs (every 1 cM in this study). Second, this mapped region is fully characterized, i.e., the significant effects are identified, along with the linkage phase between markers and QTLs, and finally the QTL segregation pattern was inferred. In diploids, a QTL can exhibit a segregation ratio of 1∶1, 1∶2:1, 3∶1 or 1∶1:1∶1. Details regarding this methodology are described by Gazaffi [27]. To perform the CIM analysis, cofactors were included in the model to control the QTLs located outside the mapping interval. For this purpose, multiple linear regression analyses using stepwise selection based on the Akaike Information Criterion were considered; a maximum of 10 cofactors was used to avoid over parameterization of the model. The threshold value for considering evidence was obtained using a 0.95 significance level and 1,000 permutations [37] according to the modification proposed by Chen and Storey [38]; i.e., the highest LOD Score peak for each linkage group was obtained, and the distribution of the second highest peak was assumed.

### Ethics Statement

We confirm that no specific permits were required for the described field studies. This work was a collaborative research developed by researchers from Edouard Michelin Plantation (Brazil), CIRAD (France), USP (Brazil) and UNICAMP (Brazil). Dr. Saulo Emilio Almeida Cardoso, researcher from Edouard Michelin Plantation in Brazil (where the field experiments were developed) is one of the coauthors of this work, and the Edouard Michelin Plantation is listed in the author addresses. Dr. Cardoso assisted in the field studies. Also, we confirm that this manuscript is a result of a basic research project, developed mainly at the university and mostly funded by public funding agencies, whose aim is to develop new knowledge and, where the generated results should be shared with the scientif and technological community through open university thesis and manuscripts in conventional scientific journals. In this sense, we would like to state that we fully adhere to all the PLOS ONE policies on sharing data and materials.

We confirm that the field studies did not involve endangered or protected species.

## Results and Discussion

### Marker Polymorphism and Segregation Analyses

Among all of the evaluated microsatellite markers, 51% (308) showed a polymorphism between the parents of the mapping population. This level of polymorphism observed in the studied population was expected because a similar level of polymorphism was previously reported in a study by Lespinasse et al. [9]. Although SSRs derived from ESTs have been shown to be less polymorphic (39%) than SSRs derived from genomic sequences (54.5%), they are present in gene-rich regions of the genome and are frequently detected at a relatively high abundance [39]. A total of 284 polymorphic markers were selected for genotyping of the 270 offspring within the mapping population.

A chi-squared test performed on the genotyped polymorphic loci revealed that 138 (48.6%) loci exhibited a segregation ratio of 1∶1, 31 (10.9%) loci exhibited a ratio of 1∶2:1, and 115 (40.5%) loci exhibited a ratio of 1∶1:1∶1. This is a very favorable scenario for building integrated linkage maps. Of the evaluated markers, 3% (9) showed distorted segregation (*P*≤0.005, used as a conservative value for the chi-squared test). The linkage analysis revealed that the markers exhibiting distorted segregation were distributed throughout the genome and were not cause distortion in the LGs (linkage groups). The low number of genomic regions showing a skewed segregation in this map is similar to the number reported for several crops, such as melon [40], but differs from the number reported for other crops, such as lettuce [41]. The deviations from the Mendelian segregation ratios detected in this study may be the result of various processes, including gametophyte selection for sub-lethal genes, i.e., genes controlling the viability of the pollen, zygotes or seedlings that are frequently located within one or more of these linkage groups [42].

### Linkage Map

An integrated genetic map was obtained using microsatellite markers. The map contains markers segregating as 1∶1:1∶1, 1∶2:1 and 1∶1 fashion. To label the linkage groups, previous maps were used as reference. The estimate map contained 284 markers assigned to 23 linkage groups and spanned a total of 2688.8 cM; the previous map developed for the rubber tree was considerably smaller [9], more informative genetic linkage was determined because it was possible to use markers with segregation ratios of 1∶1:1∶1, 1∶2:1 and 1∶1 in order to estimate an integrated map, better results were achieved. Le Guen et al. [26] and Triwitayakorn et al. [43] developed a map for the rubber tree using markers with segregation in both parents, although this map was analyzed as a double pseudo-testcross.

**Figure 1:**
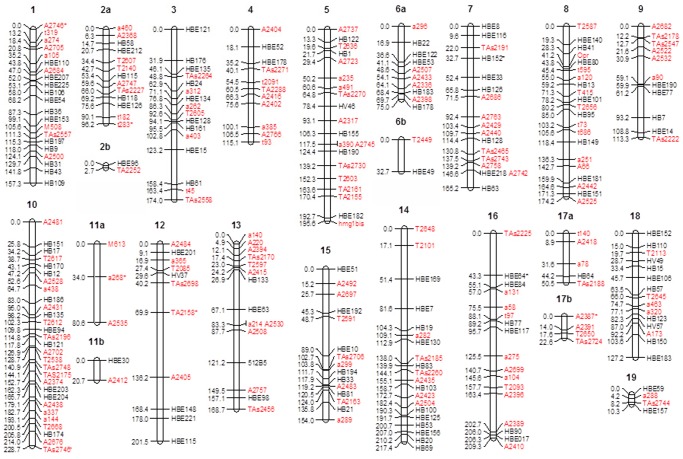
The genetic linkage map of the rubber tree. Note that markers with distorted segregation is indicated with *, the origin of the marker is indicated as follows: the HBE prefix indicates that the marker was identified by Feng et al. [28], the HV prefix indicates that the marker was identified by Gouvea et al. [41], the HB prefix indicates that the marker was identified by Souza et al. [13] and Mantello et al. [16], and the markers in red were identified by Le Guen et al. [14].

The length of each group ranged from 2.7 cM (LG2b) to 228.7 cM (LG10) (Figure 1). The distribution of the markers in each chromosome is shown in Table S1. In Figure 1, the distorted markers are indicated with an asterisk (P≤0.000172414), and the origin of each specific marker is indicated by the marker name prefix or is colored. For example, markers with the prefix HBE were identified by Feng et al. [29], markers with the prefix HV were identified by Gouvêa et al. [44], markers with the prefix HB were identified by Souza et al. [13] and Mantello et al. [16] and markers colored red were identified by Le Guen et al. [14].

The linkage groups were organized according to the numbers obtained from the map previously developed by Lespinasse et al. [9], and information from other maps (unpublished) was used to identify syntenic markers. Based on this information, the 23 groups were classified into groups numbered from 1 to 19, and groups 2, 6, 11 and 17 were further divided into subgroups “a” and “b”. The density of the markers in our map is lower (one marker for every 10 cM) than the density in the map developed by Lespinasse et al. [9] (one marker per 3 cM), although this previous map was also saturated with many AFLP markers. Additionally, the marker distribution among the linkage groups (LGs) was not uniform, which resulted in several gaps (LG 11a, 12, 13, 15 and 16). The largest gap, 66.3 cM, was observed in LG 12 (Figure 1).

One possible explanation for the observed gaps, especially for those groups that could not be linked together, is that there are fewer SSR and EST-SSR polymorphisms in these regions. For example, the published map indicates that clusters of markers for LG 6, primarily the AFLP markers, are found in certain linkage group regions; however, it is not possible to join LG 6a and 6b. AFLP clustering has been frequently reported in saturated maps for melons [40], lettuce [41] and potatoes [45] and is usually associated with the heterochromatic regions near the centromeres. Although the regions showing AFLP clustering may be indicative of the centromere positions, comprehensive cytogenetic analyses are necessary to demonstrate this association in the rubber tree.

Another possible explanation for the gaps observed in the map is that either the recombination events or mapped loci were not evenly distributed throughout the genome. The low density of markers for certain linkage groups may correspond to highly homozygous regions that have a lower recombination frequency [46].

The expected number of eighteen linkage groups for the linkage map of the rubber tree (2*n = *36) was exceeded by five linkage groups, all of which showed a low number of markers per linkage group. The small size of some of the linkage groups indicates that the additional observed linkage groups may be the result of an incomplete coverage of the genome by the marker loci. A comparison of our results with the maps published by Lespinasse et al. [9] and Le Guen et al. [26], which used several markers, indicates that the identification of 18 linkage groups is possible. Furthermore, using only SSR and EST-SSR markers, Triwitayakorn et al. [43] published a genetic linkage map in which the number of linkage groups exceeded the haploid number. These findings are consistent with the data in this study, in which the identification of 23 linkage groups suggests that different markers are required to fill the gaps between adjacent markers.

### Phenotypic Trait Analysis

The field data analysis indicated that the experiments were well conducted; this reliability is especially evident in the coefficient of variation values that ranged from 13.24 to 27.05% (Table 1). These values are relatively small considering that the experiment was performed using a perennial species and a large experimental area (7.2 ha).

**Table 1:** Mean values for girth and height growth during the summer and winter seasons, measured over a two-year period.

Traits	PB217(1)	PR255(1)	F_1_ (min – max)(1)	σ^2^g(2)	σ^2^f(2)	h^2(2)^	CV(2)
**Summer Height (SH)**	138.3	139.1	145.48 (124.4–164.2)	110.7 (27.0)	800.2	0.13	17.0
**Winter Height (WH)**	47.9	55.7	60,9 (41.45–92.57)	76.9 (14.1)	381.6	0.20	27,0
**Total Height (TH)**	191.7	197.7	205.9 (187.8–227.1)	116.7 (33.2)	1061.9	0.10	13.7
**Summer Girth (SG)**	75.4	82.4	84.5 (64.31–101.80)	65.7 (9.9)	238.9	0.27	14.1
**Winter Girth (WG)**	14.3	18.6	19,1 (12.4–31.7)	13.19 (1.8)	41.1	0.32	25.9
**Total Girth (TG)**	89.6	100.6	103.7 (78.6–127.5)	112.5 (15.2)	344.1	0.32	13.2

(1) Mean values in cm.

(2) Genotypic (σ^2^g) and phenotypic (σ^2^f) variances, heritability (h^2^) and coefficient of variation (CV). Values in parenthesis are the confidence intervals.

The phenotypic values of the parents (PB217 and PR255) as well as the progeny phenotypic segregation for all of the traits are shown in Figure S1. With the exception of the Summer Height, the phenotypic values for all of the studied traits differed between the parents. These values were higher in PR255 than in PB217 (Table 1). This difference can be attributed to the better adaptation of PR255 to cold weather, such as the winter season with suboptimal conditions for rubber tree growth, used during this study. Transgressive segregation was also observed for all of the traits evaluated. The differences in the phenotypic values analyzed for the progeny was greater than the difference between the phenotypic values of the parents (Table 1). For example, for the Summer Girth, Winter Girth and Total Girth, the progeny differences were 4.3, 3.9 and 3.4 times, respectively, greater than the phenotypic differences of the parents. A high parent heterosis is especially apparent for Height, as indicated by the mapping population distribution that is skewed to the right, showing that the average is higher than the phenotypic values of the parents.

The statistical analysis showed that the heritability varied from 0.13 to 0.32 (Table 1). A comparison between seasons indicated that the estimates for summer (Height, 0.13 and Girth, 0.27) are lower than the estimates for winter (Height of 0.20 and Girth of 0.32). This seasonal variation occurred because the environmental conditions had a greater influence on the phenotypes during the summer season. The genetic variability for Height and Girth was larger during the summer (110.7 and 65.7, respectively) than during the winter (76.9 and 13.19, respectively). This difference occurred because the environmental stress of the winter season reduced the genetic potential for growth in the genotypes; hence, the winter environment does not present an optimal climate for rubber tree growth. A heritability comparison between the traits also indicated that the values obtained for Height (0.10< h <0.20) were lower than the values obtained for Girth (0.27< h <0.32). Although similar values were reported by [47], our estimates were generally lower than previously reported values, especially with respect to plant height. Findings from a study on a eucalyptus species support the widely accepted concept that aspects of growth are under polygenic control due to the relatively low heritability of these traits [48].

In general, high correlations (Figure S2) were found when comparing the Summer Girth to the Total Girth and the Summer Height to the Total Height (0.95 and 0.75, respectively). These correlations indicate that a substantial amount of growth occurs during the summer, what was expected in our experimental conditions. The growth during the winter, however, also contributes to the total Height and Girth, as indicated by the moderate observed correlations (0.45 and 0.69, respectively). The correlations observed between the seasons for Girth (0.43) and Height (−0.22) indicate that the genotypes that performed better during the summer do not necessarily perform as well during the winter. This finding illustrates the difficulties to do rubber trees breeding in the non-traditional regions of Brazil, also referred to as escape areas. The success of a genotype during a period in which there is no environmental stress can differ from the success of that same genotype when it is exposed to a situation in which there is abiotic stress. It should also be noted that moderate weak correlations were observed between Height and Girth during the summer (0.23) and winter (0.18) seasons. These results illustrate that higher correlations were obtained when traits were compared between the seasons and indicate that the environmental influence is an important component to be considered when studying the growth traits of the rubber tree.

### QTL Mapping

**Figure 2:**
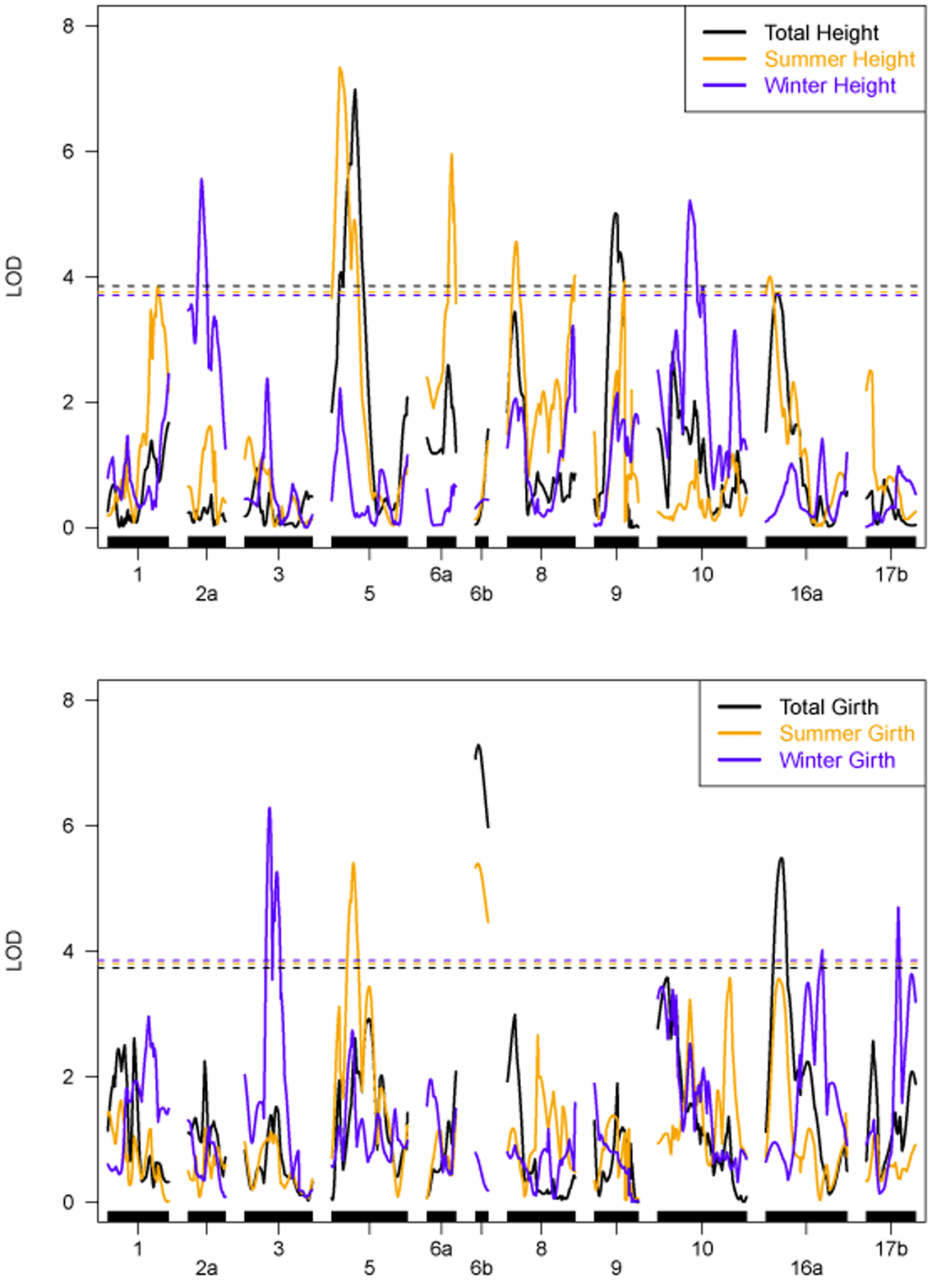
QTL mapping for height (upper panel) and girth (lower panel) for summer (orange), winter (violet) and total development (black). Note that dashed lines represents threshold values obtained with 1000 replicates.

QTL mapping was performed for Summer Height (SH), Winter Height (WH), Total Height (TH), Summer Girth (SG), Winter Girth (WG) and Total Girth (TG) by applying a composite interval mapping (CIM) model to the integrated genetic map [27]. Using this method, 18 QTLs were detected in 11 linkage groups (Figure 2; Table 2). Of the detected QTLs, 9 were identified for summer (7 for Height and 2 for Girth), 5 for winter (2 for Height and 3 for Girth) and 4 for the total values (2 for Height and 2 for Girth). The part of the phenotypic variation explained by the QTLs ranged from 2.72% to 8.97%, and the QTLs segregated according to the ratios 1∶1:1∶1, 1∶2:1, 3∶1 and 1∶1 (Table 2). To detect a QTL, a threshold value for the LOD Score was obtained based on 1,000 permutations, and these values were similar for all of the traits (3.74 for SH, 3.71 for WH, 3.85 for TH, 3.81 for SG, 3.83 for WG and 3.73 for TG).

**Table 2:** Mapped QTLs for height and girth growth during the summer and winter seasons.

QTLsname	Flanquing marquers	LG(1)	Position (cM)	Global LOD	R^2(1)^	Additive effect PB217	LOD(2)	Additive effect PR255	LOD(2)	Dominance effect	LOD(2)	Segregation
**SH.1**	HB31	1	129.73	3.85	5.51	0.6343	0.62	−1.1830	2.12	−1.0541	1.71	1∶2:1
**SH.2**	T2636-HB1	5	21.00	7.33	6.60	1.3617	2.89	1.6181	4.06	0.2631	0.11	1∶2:1
**SH.3**	A2336	6a	64.06	5.95	5.23	1.6770	4.13	0.7106	0.81	0.6478	0.66	1∶1
**SH.4**	HBE140-HB41	8	23.00	4.56	5.58	0.3421	0.14	1.8253	4.30	−0.3767	0.15	1∶1
**SH.5**	HBE151-A2525	8	174.00	4.02	3.82	1.0036	1.51	0.4135	0.26	−1.3086	2.45	1∶2:1
**SH.6**	HBE77-HB7	9	78.00	4.41	2.72	−1.1673	1.89	0.5608	0.48	−4.0434	3.69	1∶1:1∶1
**SH.7**	TAs2225-HBE64	16a	10.00	4.01	4.07	1.3488	0.76	1.5550	1.03	0.2046	0.03	1∶1
**WH.1**	T2607	2a	34.43	5.55	6.42	−1.2659	2.53	−1.2586	2.50	−0.0066	0.00	1∶2:1
**WH.2**	HB186	10	82.99	5.21	6.48	−0.4545	0.32	−1.7141	4.47	0.6631	0.63	1∶1
**TH.1**	a491	5	60.57	6.98	8.07	1.7309	4.52	0.8814	1.21	0.8265	1.08	3∶1
**TH.2**	A2532-a90	9	54.00	5.05	3.19	−0.3608	0.13	0.3308	0.15	−3.0877	4.89	1∶1
**SG.1**	a235–a491	5	56.00	5.40	5.25	0.0952	0.01	1.8027	5.13	0.2791	0.10	1∶1
**SG.2**	T2449-HBE49	6b	6.00	5.41	7.66	1.7824	3.24	−1.7277	3.46	0.3765	0.17	1∶2:1
**WG.1**	HB24-a312	3	64.00	6.28	7.39	0.3638	1.01	0.6775	3.76	0.6172	2.76	3∶1
**WG.2**	a104	16a	145.63	4.01	2.84	−0.0832	0.07	0.6368	3.77	−0.1774	0.30	1∶1
**WG.3**	HB123	17b	82.26	4.70	6.68	−0.3362	0.99	−0.1617	0.25	0.6684	4.00	1∶2:1
**TG.1**	T2449-HBE49	6b	8.00	7.32	8.97	2.8462	3.89	−2.9124	4.77	0.6612	0.26	1∶2:1
**TG.2**	TAs2225-HBE64	16a	40.00	5.49	5.14	2.2344	4.12	0.6982	0.42	−1.0117	0.78	1∶1

(1) LG indicate linkage group and R^2^ is a phenotype variation.

(2) To detect a QTL, a threshold value for the LOD Score was obtained based on 1,000 permutations, and these values were similar for all of the traits (3.74 for SH, 3.71 for WH, 3.85 for TH, 3.81 for SG, 3.83 for WG and 3.73 for TG).

For SH, 7 QTLs were identified (Table 2) in linkage groups 1 (129.73 cM), 5 (21 cM), 6a (64.06 cM), 8 (23 cM and 174 cM), 9 (78 cM), and 16 (10 cM), which explained 30.61% of the phenotype variation (R^2^). The QTL located in LG 5 had the highest peak for SH with an LOD Score of 7.33. LG 5 also had the highest peak observed for all Height traits. The phenotypic variation explaining by each QTL ranged from 2.72 (LG 9) to 6.6 (LG 5). QTLs in linkage groups 1, 5 and 8 (174 cM) had a 1∶2:1 segregation pattern, the QTLs in LGs 6a, 8 (23 cM) and 16a had a 1∶1 type of segregation pattern and QTL located in LG9 segregated in a 1∶1:1∶1 fashion. Generally, the parents contributed equally to the trait variability. Of the 7 mapped QTLs, 3 had additive effect for PB217 (LG 6a, LG 8 at 174 cM and LG 9), 3 for PR255 (LG 1, LG 8 at 23 cM and LG 16a) and one QTL had an additive effect for both parents (LG5). A dominance effect was also detected in three mapped QTLs (LG 1, LG 8 at 174 cM and LG 9). This type of result can only be verified by performing QTL mapping using an integrated map, showing the advantage of using the procedure hereby implemented.

QTL mapping for Winter Height indicated that two QTLs in linkage groups 2a (34.43 cM) and 10 (82.99 cM) explained 6.42% and 6.48% of the phenotypic variation, respectively. The former has a significant additive effect from both parents with a 1∶2:1 segregation pattern. The latter only has a significant additive effect from PR 255 and has a 1∶1 segregation pattern.

For Total Height, two additional QTLs were mapped in linkage groups 5 (60.57 cM) and 9 (54 cM), accounting for 10.64% of the phenotypic variation. The first QTL has a LOD Score of 6.98 and an R^2^ value of 8.07%. Furthermore, in agreement with its significant genetic effects and statistical similarities, this QTL has a 3∶1 segregation pattern. The second QTL has a lower LOD Score and R^2^ value (5.05 and 3.19, respectively) and has a 1∶1 segregation pattern due to its significant dominance effect.

Summer Girth has mapped QTLs in linkage group 5 (56 cM) that account for 5.25% of the phenotypic variation, and its 1∶1 segregation pattern is the result of a significant additive effect from parent PR255. Another QTL was mapped in linkage group 6b (6 cM) and has a LOD Score of 5.41, an R^2^ value of 7.66% and a 1∶2:1 segregation pattern that is the result of two similar and significant additive effects from the PR255 and PB217 parents.

Three QTLs for Winter Girth were mapped in linkage groups 3 (64 cM), 16a (145.63 cM) and 17b (82.26 cM) and account for 15.80% of the phenotypic variation. The first QTL mapped in linkage group 3 has the highest LOD Score and R^2^ value for this trait (6.28 and 7.39, respectively) and has a 3∶1 segregation pattern. This QTL segregated in a 3∶1 ratio because all of its genetic effects are significant and similar. The second QTL mapped in linkage group 16a has a LOD Score of 4.01 and a R^2^ value of 2.84%. This QTL only has a significant additive effect from PR255 and segregated in a 1∶1 ratio. The QTL mapped in linkage group 17b has a LOD Score of 4.70 and a R^2^ of 6.68. This QTL has an additive effect from PB217 and a significant dominance effect that results in a 1∶2:1 segregation pattern.

For Total Girth there were two QTLs in linkage groups 6b (8 cM) and 16a (40 cM). The first QTL in linkage group 6b has a LOD Score of 7.32 and the highest R^2^ value (8.97%). It segregates in a 1∶2:1 ratio because both of the additive effects were significant. The second QTL has a LOD Score of 5.49 and an R^2^ value of 5.14. This QTL has only one significant additive effect (PB217) that results in a 1∶1 segregation pattern.

A comparison between the summer and winter seasons showed that the QTLs detected during the summer were different from the QTLs detected during the winter for Height and Girth growth (Figure 2). Differences were also observed when comparing the QTL mapped for Winter Height versus Total Height and Winter Girth versus Total Girth. For linkage group 6b, a QTL was mapped for Summer Girth at 6 cM and another QTL was mapped for Total Girth at 8 cM, indicating that the same QTLs control both traits. Furthermore, the 1∶2:1 segregation pattern was the result of the significant additive effects from both parents.

In general, the phenotypic analysis indicated that higher correlations were found for growth between the summer and total period. This type of relationship was also observed in the QTL mapping because two QTLs could be linked for Summer Height and Total Height, and a potentially pleiotropic QTL was identified for Summer Girth and Total Girth. These results are interesting because they emphasize that when QTL mapping is conducted for the total growth period, the observed variability frequently occurs during the summer, during which rubber trees growth was maximized. Furthermore, it is interesting that no QTLs were found to occur during both the summer and a winter season, suggesting differential gene control under environmental stress. Such differential gene control would inhibit the expression of the regions responsible for growth, which causes the observed suboptimal response during the winter period. The study of growth during the winter season, however, did reveal other regions on the genetic map that control growth for this progeny. These QTLs may be important in future studies that attempt to identify specific polymorphisms that provide tolerance against cold and/or water stress.

There were QTLs that were only mapped during the summer period. The linkage group 5 has one QTL for each trait, spaced by 35 cM. The additive effect from PR255 is conserved for both traits, although the additive effect from PB217 is only present for Height. In the PR255 parent, we believe that both QTLs are linked by coupling when they show the same signal. Previous studies in *Pinus radiata* [49] and *E. globulus* [50] have shown that a single co-located QTL affects traits, and there are several independent QTLs for growth. Taken together, these findings suggest that the growth rate and wood property traits could be simultaneously improved through a proper breeding program.

A direct comparison of the regions mapped in different studies on rubber trees is challenging because neither the investigated markers and the populations and the traits nor the methodologies are comparable. For example, Lin et al. [51] proposed a QTL mapping model for outbred species using genetic maps with molecular markers showing different segregation patterns. Our approach was different because we did not model the linkage phase between QTL and markers with parameters from the likelihood, thus reducing substantially the complexity of EM algorithm. In our model the linkage phase between QTL and markers was inferred by the interpretation of the sign of additive effects, and the QTL segregation was inferred for mapped QTL. One of the advantages of our approach was the possibility to include cofactors (previously selected by multiple linear regressions) in the model, increasing the statistical power [36], [52]. Another difference of our model was the usage of multipoint probabilities based on Hidden Markov Models [23], [24], which is useful specially for interval flanked by not fully informative markers.

Some previous QTL mapping studies in rubber trees have been performed to characterize *Microcyclus ulei* resistance [10], [25], [26]. To our knowledge, this present work is the first QTL mapping study to use an integrated genetic map for the rubber tree. This method allowed for the identification of QTLs with different segregation patterns as well as QTLs with a dominance effect, which was not possible with the approaches used in previous studies. Here, ten (55.5%) of the 18 mapped QTLs has a segregation pattern of either 1∶1:1∶1, 1∶2:1 or 3∶1, which may have contributed to the R^2^ values obtained here.

The parents PR255 and PB217 had contrasting and complementary phenotypes. The significant additive effects detected in this study indicate that both parents contributed equally to the polymorphisms in the investigated traits. Among the significant marginal effects that were identified, 11 were associated with PB217 and 12 were associated with PR255. The results obtained using QTL mapping provides novel insights into the genetic control of growth characteristics in the rubber tree. Among the 18 QTLs that were detected, 7 (38.8%) are the result of dominance effects. Although additive effects are the primary cause of the polymorphisms in the progeny, the dominance effects play an important role in the genetic architecture of these traits.

The results hereby presented indicate that complex traits in rubber trees are controlled by many genes and the individual effect of one of these genes on the phenotype is small. Furthermore, these results indicate that broad genome-wide searches will be required to identify all of the genes that control a complex trait as well as all of the segregating variation in a population [53].

Height and Girth are considering as quantitative traits and are influenced by many variables, finding QTLs explaining these changes is difficult. The low heritability is also difficult to identify QTLs, thus, few regions are detected, so the chance to detect QTLs in commons between summer and winter is also reduced.

The approach used here is informative and provides interesting insights about growth traits in rubber tree. This could be combined in the future with ideas related to functional mapping [54]–[57]. By considering a series of repeated measures collected over time, these authors suggested a logistic model for growth pattern. This would allow a better understanding of QTL expression over time and will be considered for future studies.

The QTLs identified in this work, particularly those related to phenotypic variation during the winter season, can be used to begin investigating the potential for marker-assisted selection, especially when the objective of a breeding program is to obtain phenotypes adapted to sub-tropical climate areas. Studying the behavior of plants in winter will be very interesting to find polymorphism that allows better adaptation to winter, for example, some resistance to cold and dry, than finding genotypes that develop in the cold with the same intensity as they would in the summer. These studies are possible because the winter climate (April to October) in the location where these experiments were performed is dry and cold compared to the summer climate (Figure S3); thus, the fungus *M. ulei* is not well adapted to this type of climate. Further studies using other crosses are required to identify additional QTLs that affect frost tolerance in *H. brasiliensis*. The long developmental cycle of rubber trees presents a challenge to QTL mapping, and the size of the experiment is a limiting factor for establishing mapping populations in different environments. Therefore, further studies investigating traits associated with the development of these plants are necessary for the selection of genotypes that are adapted to sub-tropical climate areas.

## Supporting Information

Figure S1 Distribution of the phenotypic data for the F1 population and the genitor (PB217 and PR255).
(TIF)

Figure S2 Genotypic correlation coefficients and dispersion of the phenotypic data. Genotypic correlation coefficients and dispersion of the phenotypic data for each trait measured during the summer and winter seasons. (*significant at the 5%; **significant at the 1%; ***significant at 5% of global level - Bonferroni correction for multiple tests).
(TIF)

Figure S3 The average temperatures and precipitation. The average temperatures (maximum and minimum) and precipitation for the years 2006 to 2009 in Itiquira-MS.
(TIF)

Table S1 The distribution and type of SSR and EST-SSR markers in the linkage groups.
(TIF)
