# Supplementary figures and images for: QTL Mapping of Growth-Related Traits in a Full-Sib Family of Rubber Tree (Hevea brasiliensis) Evaluated in a Sub-Tropical Climate
Source: PLoS One. 2013 Apr 19;8(4):e61238. doi: 10.1371/journal.pone.0061238 (PMC3631230; doi:10.1371/journal.pone.0061238)

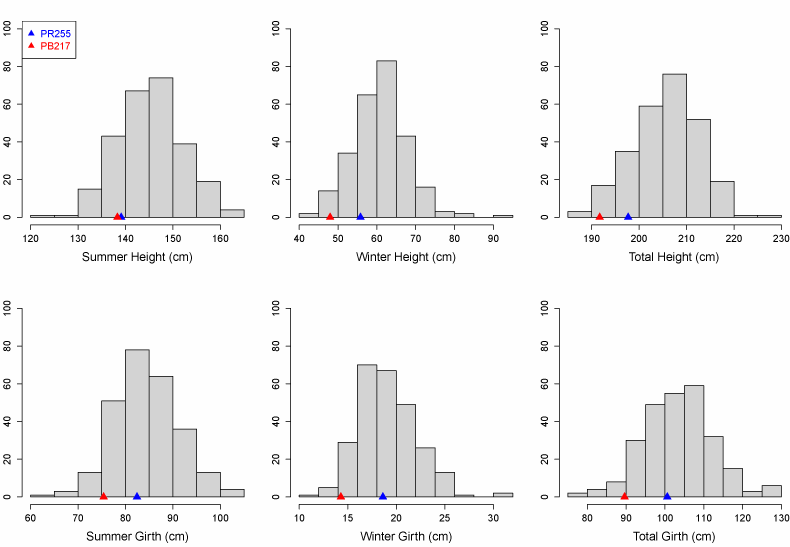

Supplement: Figure S1 — Distribution of the phenotypic data for the F1 population and the genitor (PB217 and PR255). (TIF) [file pone.0061238.s001.tif]

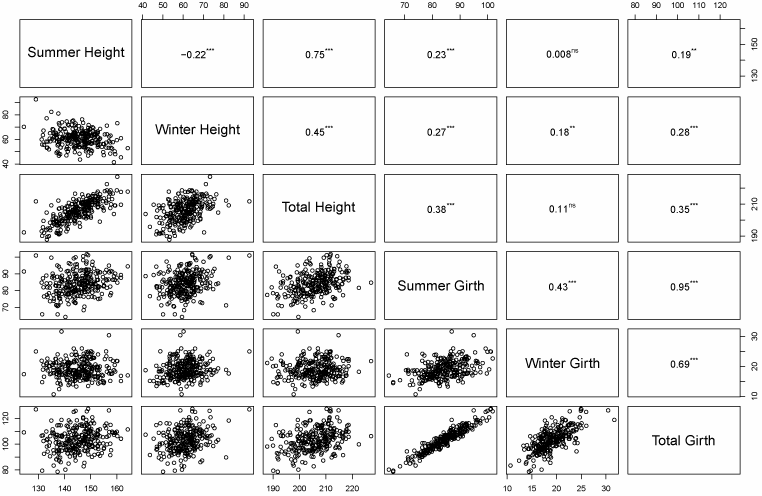

Supplement: Figure S2 — Genotypic correlation coefficients and dispersion of the phenotypic data. Genotypic correlation coefficients and dispersion of the phenotypic data for each trait measured during the summer and winter seasons. (*significant at the 5%; **significant at the 1%; ***significant at 5% of global level - Bonferroni correction for multiple tests). (TIF) [file pone.0061238.s002.tif]

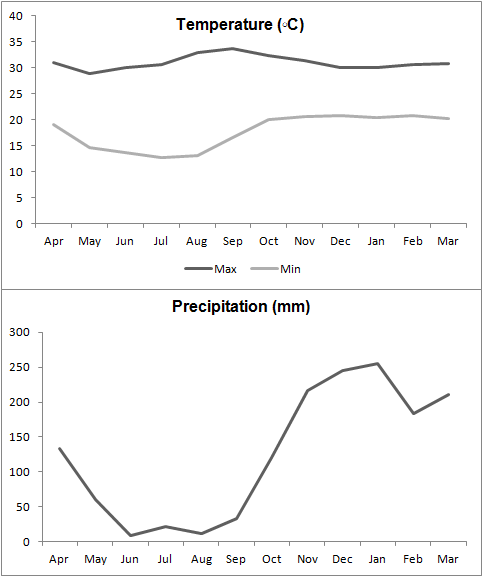

Supplement: Figure S3 — The average temperatures and precipitation. The average temperatures (maximum and minimum) and precipitation for the years 2006 to 2009 in Itiquira-MS. (TIF) [file pone.0061238.s003.tif]

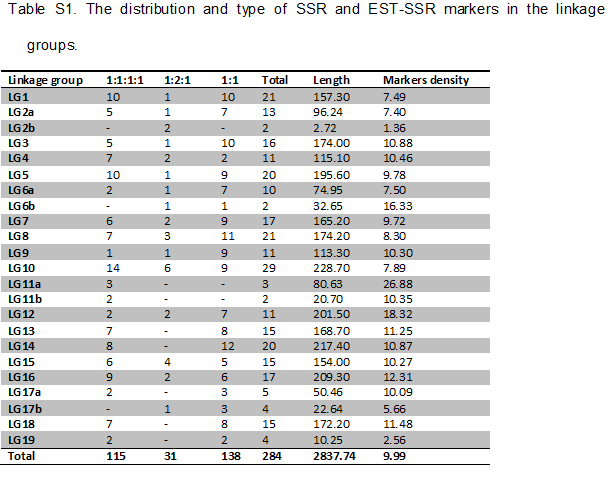

Supplement: Table S1 — The distribution and type of SSR and EST-SSR markers in the linkage groups. (TIF) [file pone.0061238.s004.tif]
